# Radiofrequency Ablation for Atrial Arrhythmia After Esophagectomy

**DOI:** 10.1016/j.jaccas.2025.106498

**Published:** 2026-03-04

**Authors:** Yunyi Li, Jiongchao Guo, Xiaofeng Hu, Weifeng Jiang, Yang Liu, Xu Liu, Shaohui Wu

**Affiliations:** Shanghai Chest Hospital, Shanghai Jiao Tong University, Shanghai, China

**Keywords:** atrial arrhythmia, esophagectomy, radiofrequency ablation

## Abstract

**Background:**

New-onset atrial fibrillation is common after esophagectomy, whereas atrial premature contractions and atrial tachycardia are relatively uncommon.

**Case Summary:**

A 73-year-old man developed paroxysmal palpitations after esophagectomy with gastric pull-up reconstruction for esophageal cancer. Symptoms persisted despite oral medication and worsened over the past year. The postadmission electrocardiogram revealed frequent atrial premature contractions and atrial tachycardia. Cardiac electrophysiology study and radiofrequency ablation were performed. Mapping indicated a close anatomical relationship between the gastric conduit and the atrial premature contraction focus. No arrhythmia recurrence was observed during follow-up.

**Discussion:**

This case highlights premature atrial complexes and atrial tachycardia after esophagectomy, refractory to drugs but successfully treated with ablation. The anatomical proximity of the gastric conduit to the ectopic focus suggests local mechanical irritation as a potential mechanism.

**Take-Home Messages:**

New-onset atrial arrhythmias after esophagectomy may involve mechanical irritation from adjacent structures. When there is an inadequate response to pharmacotherapy, radiofrequency ablation represents an effective alternative.

## Graphical abstract

**Figure:**
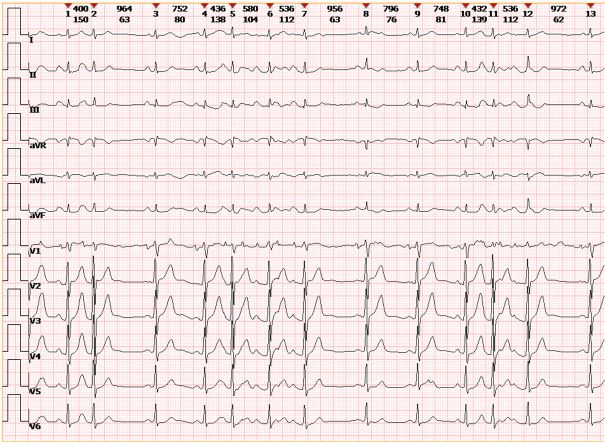

## History of Presentation

A 73-year-old man was admitted to the hospital for paroxysmal palpitations lasting for 10 years, which had exacerbated in the past year. An electrocardiogram (ECG) performed at a community hospital confirmed frequent premature atrial complexes (PACs) and atrial tachycardia. Despite treatment with anticoagulant and antiarrhythmic agents (metoprolol 25 mg twice daily and propafenone 150 mg 3 times daily), the patient’s symptomatic palpitations persisted. Consequently, he was referred to our hospital for further treatment.

**Take-Home Messages**

• For new-onset atrial arrhythmias after esophagectomy, the underlying mechanism may be associated with local mechanical irritation.
• Such arrhythmias are often refractory to pharmacological therapy, and radiofrequency ablation may be considered as a treatment option.

## Past Medical History

The patient underwent esophagectomy with gastric pull-up reconstruction for esophageal cancer 10 years ago. Follow-up computed tomography scans have repeatedly identified a right-sided pleural effusion, with no evidence of esophageal cancer recurrence or metastasis. The patient has a 5-year history of type 2 diabetes mellitus, which is currently managed with oral medication and satisfactory glycemic control. There was no history of cardiac arrhythmias before esophagectomy.

## Differential Diagnosis

The differential diagnosis includes sinus tachycardia, paroxysmal supraventricular tachycardia, atrial flutter, atrial fibrillation, supraventricular tachycardia with aberrant conduction, and ventricular tachycardia.

## Investigation

**Figure 1:**
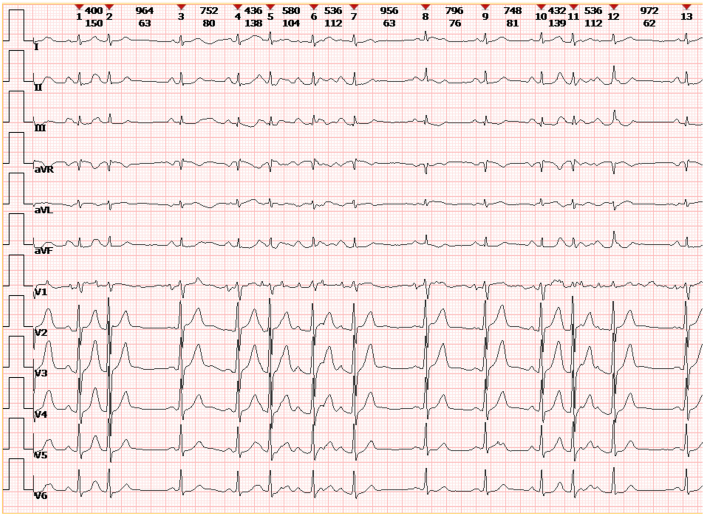
Electrocardiogram on Admission The electrocardiogram showed frequent premature atrial complexes with atrial tachycardia.

After admission, relevant ancillary investigations were completed. The patient’s vital signs were monitored and remained stable, with a temperature of 36.2 °C, a blood pressure of 112/74 mm Hg, a respiratory rate of 12 breaths/min, and a mean heart rate of 90 beats/min. A 12-lead ECG revealed PACs coexisting with atrial tachycardia (Figure 1). Biochemical testing showed markedly elevated N-terminal pro–B-type natriuretic peptide levels (760 ng/L), suggesting potential cardiac strain.

Transthoracic echocardiography demonstrated mild-to-moderate mitral regurgitation with aortic valve calcification. The left ventricular ejection fraction was 61%. The remaining cardiac structures were unremarkable, and both left ventricular systolic and diastolic functions were normal.

## Management

All antiarrhythmic drugs were discontinued for at least 5 half-lives before the initiation of the procedure. After the exclusion of relevant contraindications, the patient was scheduled to undergo radiofrequency catheter ablation.

**Figure 2:**
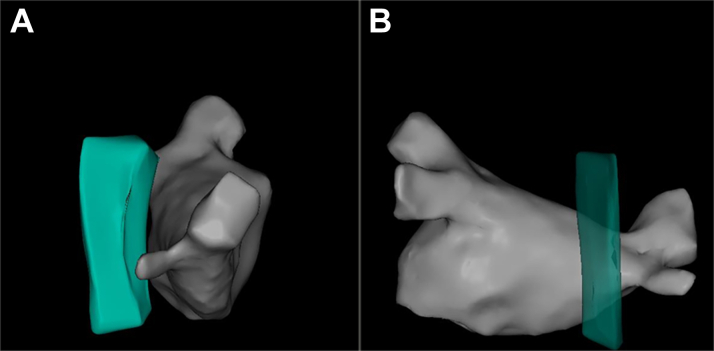
Relative Position Between the Pulmonary Veins and the Gastric Conduit Intracardiac echocardiography–based 3-dimensional reconstruction model shows the relative position between the pulmonary veins and the gastric conduit: (A) right lateral view and (B) posteroanterior view.

**Figure 3:**
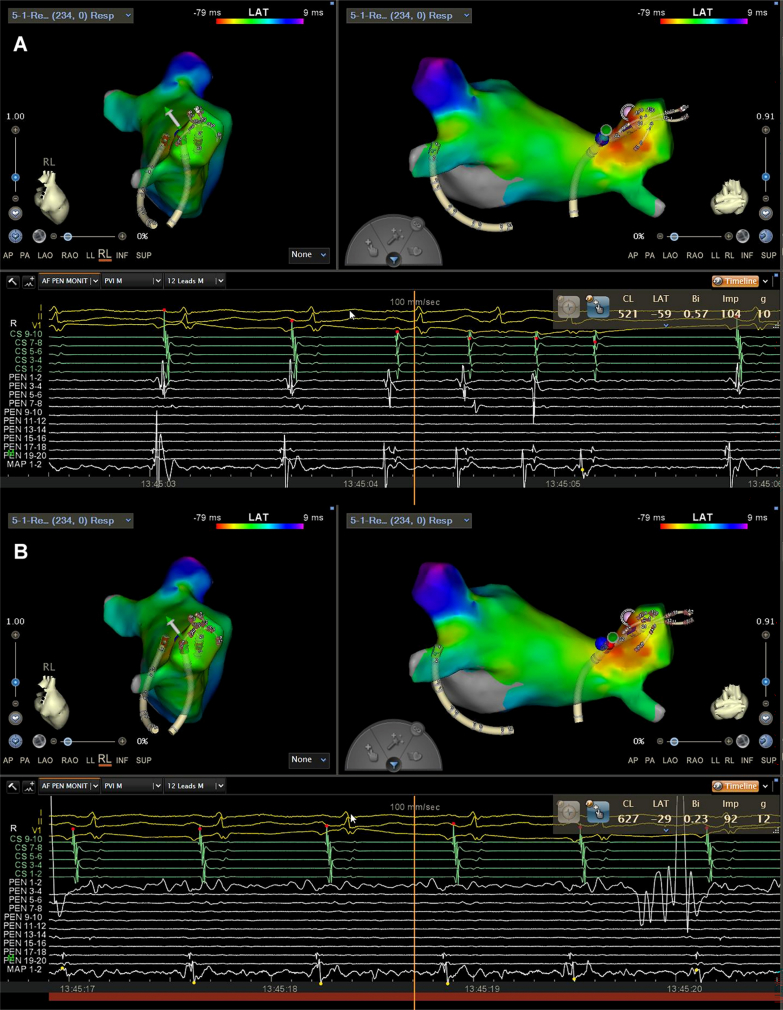
Electrocardiographic Tracings Before and After Catheter Ablation (A) Before ablation of the blue sites, atrial arrhythmias persisted. (B) Sinus rhythm was successfully restored immediately after the delivery of radiofrequency energy to the blue sites.

After routine disinfection, draping, and local anesthesia of bilateral femoral veins, an 11-F short sheath was inserted via puncture of the left femoral vein. A CartoSound ultrasound catheter (Biosense Webster) was then advanced through the venous sheath to construct a 3-dimensional cardiac model and rule out thrombus in the left atrium and left atrial appendage. A decapolar coronary sinus mapping electrode was placed via the left femoral venous sheath. Two 8.5-F Swartz sheaths (Abbott) were advanced through the right femoral vein. After transseptal puncture, heparinization was administered (100 U/kg, with activated clotting time maintained at 300-350 seconds), and a contrast agent was injected for bilateral pulmonary venography. Subsequently, a PentaRay star-shaped mapping catheter (Biosense Webster) and an STSF pressure-sensing open-irrigated ablation catheter (Biosense Webster) were advanced into the left atrium through the Swartz sheaths for left atrial 3-dimensional mapping and substrate mapping. Electrophysiological mapping revealed a relatively preserved left atrial substrate, with premature atrial contractions originating from the posterosuperior area of the right pulmonary vein antrum, located adjacent to the gastric conduit (Figure 2; Video 1). Circumferential pulmonary vein isolation was subsequently performed using an open-irrigated ablation mode at 40 W. After successful electrical isolation of bilateral pulmonary veins, atrial premature contractions and atrial tachycardia were eliminated (Figure 3). Burst pacing from the atrium failed to induce any arrhythmias, and no pulmonary vein potential recovery was observed during the monitoring period, thus concluding the procedure.

## Outcome and Follow-Up

The patient’s postoperative ECG revealed sinus rhythm. He was discharged on the second postoperative day without any in-hospital adverse events or recurrence of atrial tachycardia. During the 2-month follow-up period, he remained free from PACs or atrial tachycardia episodes.

## Discussion

New-onset postoperative atrial fibrillation (POAF) is one of the most frequent complications after esophagectomy. Previous studies have reported that the incidence of POAF after esophagectomy is approximately 16.5%. Not only does POAF function as a clinical harbinger of other complications, but it also demonstrates a clear temporal correlation with life-threatening conditions such as anastomotic leakage. Moreover, POAF is a significant marker for prolonged hospital stay, heightened reoperation risk, and increased postoperative mortality.^1^^,^^2^ However, chronic atrial arrhythmias persisting for a decade after esophagectomy have rarely been reported.

In this case report, we present a patient who developed PACs and atrial tachycardia after esophagectomy with gastric pull-up reconstruction for esophageal cancer. The patient underwent esophagectomy for esophageal cancer 10 years ago. Postoperatively, he developed palpitations and was diagnosed with paroxysmal atrial fibrillation. Although his atrial fibrillation symptoms improved with pharmacological treatment, palpitations have worsened over the past year. During this hospital period, no documentation of atrial fibrillation was obtained; however, ECG monitoring ultimately diagnosed PACs coexisting with atrial tachycardia. We propose that atrial tachycardia may represent a late complication resulting from the further progression of POAF after esophagectomy. Previous studies have demonstrated that prophylactic administration of amiodarone after esophagectomy can reduce the incidence of POAF.^3^ However, in the present case, we recommend radiofrequency catheter ablation for patients whose symptoms remain refractory to pharmacological therapy and who have no contraindications for the procedure.

During the electrophysiology study, we observed that the gastric conduit was immediately adjacent to the left atrium and in close proximity to the ectopic foci. Previous studies have reported that mechanical stimulation induced by esophageal distension can directly compress or exert traction on the left atrial wall or myocardium near the pulmonary vein orifices.^4^^,^^5^ Such mechanical stretch may elicit local atrial depolarization, thereby acting as a trigger for PACs or atrial tachycardia. Furthermore, esophageal distention may activate the sympathetic nervous system, thereby enhancing atrial automaticity and potentially initiating ectopic electrical activity.^6^ Using intracardiac echocardiography, we observed that the patient’s gastric conduit was notably dilated compared with a normal esophagus. Because pharmacological therapy failed to significantly alleviate his palpitations, we speculate that both direct and indirect mechanical effects of the gastric conduit on the atria may constitute the underlying mechanism of his frequent palpitations.

We acknowledge several limitations in this case. First, our findings are constrained by the inherent limitations of the case report format. Specifically, case reports are typically selected for their educational value or unique clinical features, thus introducing selection bias; the described patient characteristics and outcomes may not be representative of a broader patient population. Observations derived from a single patient cannot establish causality or be generalized to a broader population. Second, the follow-up period was relatively short. A longer follow-up duration is required to evaluate the long-term efficacy of radiofrequency ablation and potential late adverse effects in managing postoperative atrial arrhythmias after esophagectomy. Although ablation therapy may represent a promising intervention for arrhythmia prevention in this context, further studies are needed to validate our conclusions

## Conclusions

For patients who develop PACs or atrial tachycardia after esophagectomy for esophageal cancer, a careful differential diagnosis is essential. If symptoms remain refractory to pharmacological therapy, radiofrequency catheter ablation should be considered as a therapeutic option.

## Funding Support and Author Disclosures

This work was supported by the Project of the Intramural Incubation Program, Shanghai Chest Hospital (Grant No. 20234IIT-Q006). The authors have reported that they have no relationships relevant to the contents of this paper to disclose.

## Appendix

Video 1 Visualization of the Gastric Conduit by Intracardiac Echocardiography
